# Rapid Action of Aldosterone on Protein Levels of Sodium-Hydrogen Exchangers and Protein Kinase C Beta Isoforms in Rat Kidney

**DOI:** 10.1155/2017/2975853

**Published:** 2017-10-22

**Authors:** Somchit Eiam-Ong, Mookda Chaipipat, Krissanapong Manotham, Somchai Eiam-Ong

**Affiliations:** ^1^Department of Physiology, Faculty of Medicine, Chulalongkorn University, Bangkok 10330, Thailand; ^2^Department of Pathology, Faculty of Medicine, Chulalongkorn University, Bangkok 10330, Thailand; ^3^Department of Medicine, Lerdsin General Hospital, Bangkok 10500, Thailand; ^4^Department of Medicine (Division of Nephrology), Faculty of Medicine, Chulalongkorn University, Bangkok 10330, Thailand

## Abstract

Previous *in vitro* studies demonstrated that aldosterone rapidly activates sodium-hydrogen exchangers 1 and 3 (NHE 1 and 3). *In vitro* investigations revealed that protein kinase C (PKC) regulates NHE properties. We previously demonstrated that aldosterone rapidly enhances PKC*α* protein abundance in the rat kidney. There are no reports of renal PKC*β* (I and II) protein levels related to the regulation by aldosterone. There are also no *in vivo* data regarding the rapid effects of aldosterone on renal protein levels of NHE (1 and 3) and PKC*β* (I and II), simultaneously. In the current study, rats received normal saline solution or aldosterone (150 *μ*g/kg BW, i.p.). After 30 minutes, abundance and immunoreactivity of these proteins were determined by Western blot analysis and immunohistochemistry, respectively. Aldosterone increased NHE1 and NHE3 protein abundance to 152% and 134%, respectively (*P* < 0.05). PKC*β*I protein level was enhanced by 30%, whereas PKC*β*II declined slightly. Aldosterone increased NHE protein expression mostly in the medulla. PKC*β*I immunostaining intensity was increased in the glomeruli, renal vasculature, and thin limb of the loop of Henle, while PKC*β*II was reduced. This is the first *in vivo* study to simultaneously demonstrate that aldosterone rapidly elevates PKC*β*I and NHE (1 and 3) protein abundance in the rat kidney. Aldosterone-induced NHE (1 and 3) protein levels may be related to PKC*β*I activation.

## 1. Introduction

The role of aldosterone on sodium reabsorption has been ascribed to a genomic mechanism via binding to intracellular receptors. Then, the aldosterone-receptor complex is transferred to the nucleus, where it acts as a transcriptional regulator [1]. For nongenomic effects, aldosterone exhibits its action with a rapid onset (≤30 minutes) and is insensitive to inhibitors of transcription and translation [2–5]. The rapid actions of aldosterone on protein kinase C beta (PKC*β*) and sodium-hydrogen exchanger (NHE) properties have not been documented *in vivo*.

Aldosterone has been shown to stimulate NHE1 activity in cortical collecting duct (CCD) cells [6]. Aldosterone induced NHE3 surface expression through the activation and trafficking of NHE3 [7]. *In vitro* studies demonstrated that PKC increases NHE activity [6, 8, 9]. Aldosterone treatment caused the rapid increase of PKC*α* protein abundance in CCD cells [10]. In addition, we previously reported that aldosterone rapidly increases PKC*α* protein abundance and immunoreactivity in the rat kidney [11]. There are no investigations regarding the rapid effects of aldosterone on the simultaneous renal protein abundance and localization of NHE (1 and 3) and PKC*β* (I and II).

Currently, no *in vivo* study has examined whether aldosterone alters the protein abundance and localization of PKC*β* (I and II) and NHE (1 and 3) in a rapid pathway in the rat kidney. Therefore, the present study aims to investigate this regard. At 30 minutes after aldosterone injection, the protein abundance of PKC*β* (I and II) and NHE (1 and 3) was measured by Western blot analysis, and the localization and distribution of these proteins were examined with an immunohistochemical method.

## 2. Materials and Methods

### 2.1. Animal and Experimental Protocols

Male Wistar rats weighing 200 to 240 g (National Center of Scientific Use of Animals, Mahidol University, Nakornpathom, Thailand) were given conventional housing and diet. All animal protocols were approved by the Ethics Committee of Research, Chulalongkorn University (permit number IRB 03/56). The serum creatinine of each rat should be <1 mg/dL [11–13]. The rats were divided into two groups (*n* = 8/group): sham (normal saline solution; NSS: 0.5 mL/kg BW by intraperitoneal injection, i.p.) and Aldo (aldosterone 150 *μ*g/kg BW, diluted in NSS, i.p.; Sigma, St. Louis, MO, USA) [11–13]. We used this dose as previously performed in studies on the rapid actions of aldosterone on the protein levels of upstream/downstream mediators [12, 13], PKC*α* and *α*_1_-Na^+^/K^+^-ATPase [11]. Therefore, in the present investigation, we further examine the effect of this dose on PKC*β* (I and II) and NHE (1 and 3) protein levels.

On the date of the experiment, 30 minutes following injection of NSS or aldosterone, the rats were anesthetized with thiopental (100 mg/kg BW, i.p.) [11–13]. Plasma samples collected from the abdominal aorta were stored at −80°C until use for the measurement of aldosterone levels by a radioimmunoassay kit (Aldo-Riact; CIS Bio International, Gif-sur-Yvette, France). Blood and urine chemistry were measured by an indirect method (Model CX3; Beckman, Krefeld, Germany). The kidneys were removed, and half of each kidney was fixed in liquid nitrogen, and then stored at −80°C until use for the measurement of PKC*β* (I and II) and NHE (1 and 3) protein abundance by Western blot analysis. The other half of the renal tissue was fixed in 10% paraformaldehyde, subjected to tissue processing by an automate tissue processor (Shandon Citadel 2000, Thermo Scientific, Rockford, IL, USA), and embedded in paraffin wax for localization and distribution of these proteins by immunohistochemistry [11–13].

### 2.2. Western Blot Analysis

The renal tissue samples were homogenized on ice with a homogenizer (IKA, T25 Basic, Selangor, Malaysia) in homogenizing buffer [20 mM Tris-HCl; pH 7.5, 2 mM MgCl_2_, 0.2 M sucrose, and 5% (*v*/*v*) protease inhibitor cocktail (Sigma)]. To remove crude debris, the kidney tissues were homogenized and centrifuged at 12,000*g* (Biofuge PrimoR, Heracus, Germany) for 20 minutes at 4°C. The supernatant parts were collected for homogenate samples [11–13]. To harvest the plasma membrane, the supernatant was further centrifuged at 17,000*g* for 20 minutes at 4°C [11, 14]. The pellet was resuspended in buffer. The total protein concentrations of both homogenate samples and plasma membranes were measured with Bradford protein assay reagent (Pierce, Rockford, IL, USA) following the manufacturer's protocol. The measurement of protein abundance was performed as previously described [11–13]. Proteins were resolved on 8% sodium dodecyl sulfate polyacrylamide gel electrophoresis (SDS-PAGE) for PKC*β* (I and II), NHE (1 and 3), or *β*-actin and blotted onto nitrocellulose membranes (Bio-Rad, Hercules, CA, USA). The membranes were incubated with a primary monoclonal antibody to PKC*β*I (E-3: sc-8049; 1 : 1000; Santa Cruz Biotechnology, Dallas, CA, USA) [15], PKC*β*II (Y125: ab32026; 1 : 1000; Abcam, Cambridge, UK) [16], NHE1 (54: sc-136239; 1 : 500; Santa Cruz Biotechnology) [17], NHE3 (19F5: sc-58636; 1 : 1000; Santa Cruz Biotechnology) [18], or *β*-actin (Santa Cruz Biotechnology), followed by the respective horseradish peroxidase-linked secondary antibody (Bio-Rad). Immunoreactive proteins were detected by chemiluminescence (SuperSignal West Pico kit; Pierce) and documented using a molecular imager ChemiDoc XRS system (Bio-Rad Laboratories). The relative protein levels of PKC*β* (I and II) and NHE (1 and 3) in each sample were present as a percentage of the control normalized to its *β*-actin content.

### 2.3. Immunohistochemical Study

Detection of protein localization was performed as previously described [11–13]. Paraffin-embedded kidney sections were cut to 4 *μ*m in thickness. The slides were deparaffinized and endogenous peroxidase was blocked by treatment with 3% H_2_O_2_. The sections were incubated with the primary antibody to PKC*β*I (1 : 400; Santa Cruz Biotechnology), PKC*β*II (1 : 400; Abcam), NHE1 (1 : 100; Santa Cruz Biotechnology), or NHE3 (1 : 200; Santa Cruz Biotechnology) at 4°C overnight, followed by the respective horseradish peroxidase-linked secondary antibody (Bio-Rad), and then reacted with 3,3′-diaminobenzidine (DAB) solution (Sigma). As a negative control, the primary antibody was omitted, resulting in negative staining. In a blinded manner, three pathologists independently scored the staining intensity on a semiquantitative five-tiered grading scale from 0 to 4 (0 = negative; 1 = trace; 2 = weak; 3 = moderate; and 4 = strong) as previously described [11–13, 19].

### 2.4. Statistical Analyses

The results of renal PKC*β* (I and II) and NHE (1 and 3) protein abundance were expressed as the mean ± SD. Statistical differences between the groups were assessed by an ANOVA (analysis of variance) with a post hoc comparison by Tukey's test when appropriate. A *P* value of <0.05 was considered statistically significant. Statistical tests were analyzed using SPSS program version 22.0 (SPSS Inc., Chicago, IL, USA). The median staining intensity (score) of renal PKC*β* (I and II) and NHE (1 and 3) protein levels was presented as previously described [11–13].

## 3. Results

### 3.1. Blood and Urine Chemistry

As shown in Table 1, plasma aldosterone levels significantly increased in the Aldo group compared with the sham group (sham = 1315.11 ± 10.22 pmol/L; Aldo = 6234.33 ± 92.88 pmol/L; *P* < 0.001). No significant changes in plasma sodium, potassium, chloride, bicarbonate, creatinine, or blood urea nitrogen were observed between the sham and Aldo groups. Aldosterone did not significantly alter the ratio of plasma sodium to potassium compared with the sham group (sham = 41.27 ± 3.64; Aldo = 39.87 ± 2.94; *P* = 0.74). In addition, the ratio of urinary sodium to potassium in the Aldo group (0.32 ± 0.02; *P* = 1.00) was comparable to that in the sham group (0.32 ± 0.02).

### 3.2. Effect of Aldosterone on Renal PKC*β* (I and II) Protein Abundance

**Figure 1:**
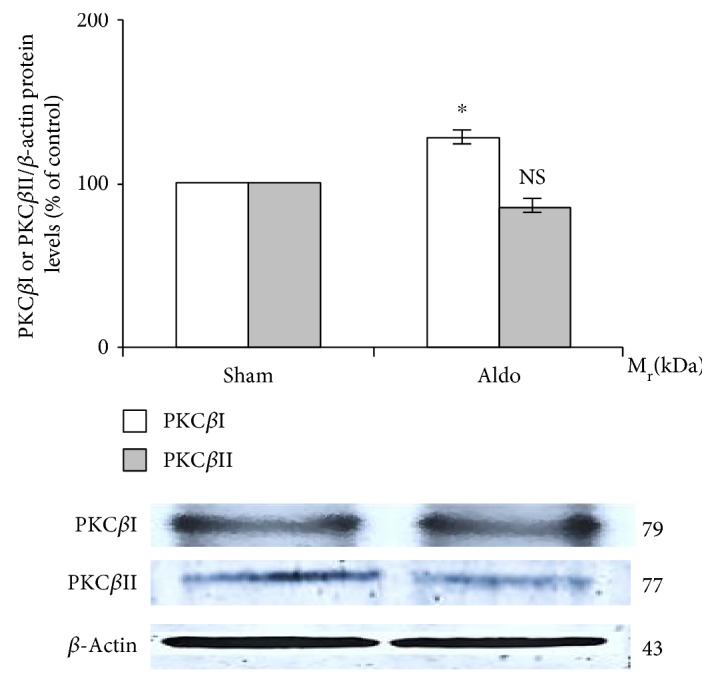
Western blot analysis of renal PKC*β*I and PKC*β*II protein abundance in the sham and Aldo groups. Histogram bars show the densitometric analyses ratios of PKC*β*I, or PKC*β*II to *β*-actin intensity, and the representative immunoblot photographs are present. Data are means ± SD of 8 independent experiments. ^∗^*P* < 0.05 compared with the sham group.

By Western blot analysis (Figure 1), the protein levels of PKC*β*I (79 kDa) and PKC*β*II (77 kDa) were assessed. Aldosterone enhanced the protein abundance of renal PKC*β*I in the sham group from 100% to 127 ± 7% (*P* < 0.05), but PKC*β*II protein tended to decline (to 85 ± 8%, *P* = 0.17) (*n* = 8/group).

### 3.3. Effect of Aldosterone on Renal NHE (1 and 3) Protein Abundance

**Figure 2:**
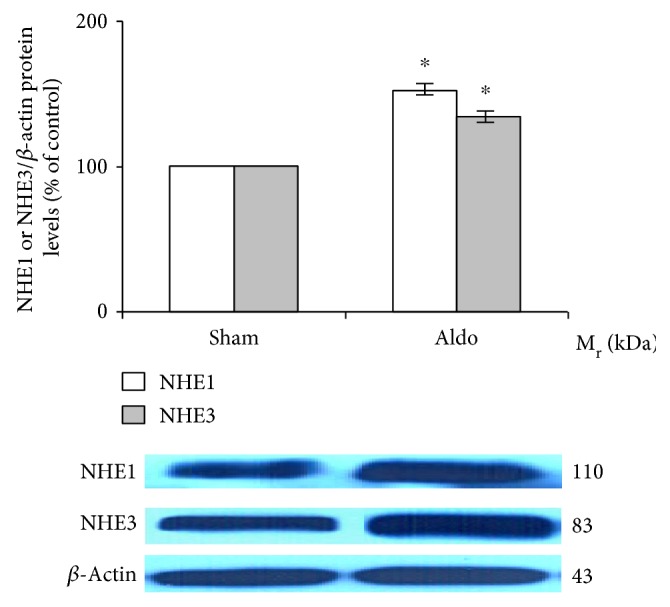
Western blot analysis of renal NHE1 and NHE3 protein abundance in the sham and Aldo groups. Histogram bars show the densitometric analyses ratios of NHE1 or NHE3 to *β*-actin intensity, and the representative immunoblot photographs are present. Data are means ± SD of 8 independent experiments. ^∗^*P* < 0.05 compared with the sham group.

The protein levels of NHE1 (110 kDa) and NHE3 (83 kDa) were measured in the rat kidney by Western blot analysis. As shown in Figure 2, aldosterone significantly elevated the protein abundance of NHE1 (sham = 100%; Aldo = 152 ± 8%, *P* < 0.05) and NHE3 (sham = 100%; Aldo = 134 ± 8%, *P* < 0.05) (*n* = 8/groups).

### 3.4. Effect of Aldosterone on Renal PKC*β*I Protein Localization

**Figure 3:**
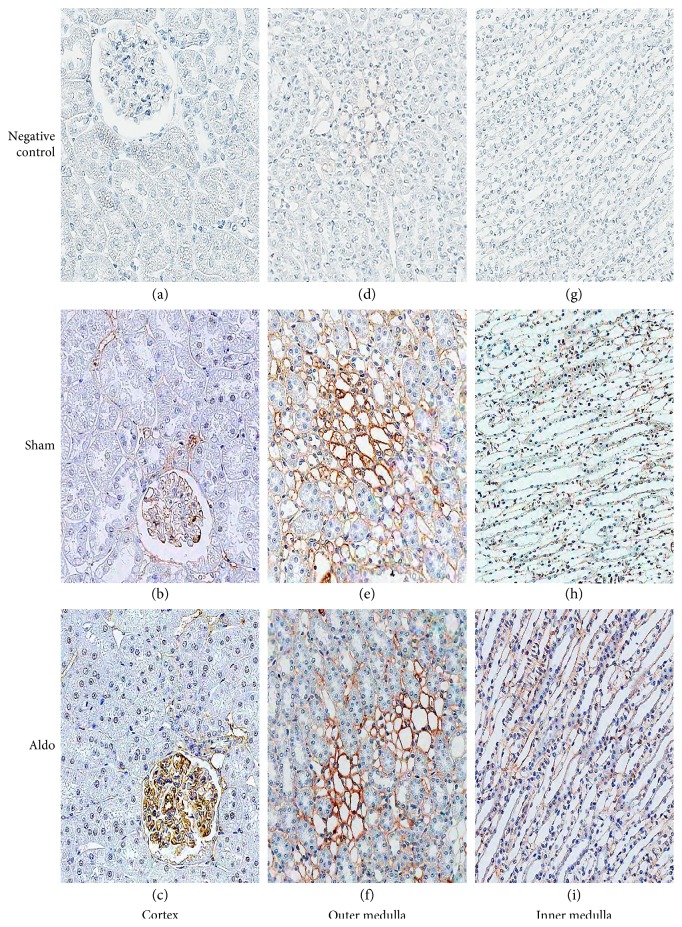
Representative immunohistochemical staining micrographs of renal PKC*β*I protein localization in the cortex (a–c), the outer medulla (d–f), and the inner medulla (g–i) from sham (b, e, and h) and Aldo (c, f, and i) (*n* = 5/group). Negative controls (a, d, and g). Original magnification, ×400 (a–c) and ×200 (d–i).

The protein localization of PKC*β*I in the cortex in the sham group is demonstrated in Figure 3, b and Table 2. The immunoreactivity was weak in the glomerulus and the peritubular capillary (Pcap), but no staining was noted in the proximal convoluted tubule (PCT), the distal convoluted tubule (DCT), or the CCD. Aldosterone increased the intensity score only in the glomerulus to moderate level (Figure 3, c). In the outer medulla (OM), aldosterone elevated the intensity score from 3 to 4 in the vasa recta (VR) and in the thin limb of the loop of Henle (tLH), but staining in the thick ascending limb of the LH (TALH) and the medullary CD (MCD) did not change (Figure 3, f and Table 2). In the inner medulla (IM), immunoreactivity was enhanced by aldosterone to weak levels in the VR and the tLH, but the intensity score in the MCD did not change (Figure 3, i).

### 3.5. Effect of Aldosterone on Renal PKC*β*II Protein Localization

**Figure 4:**
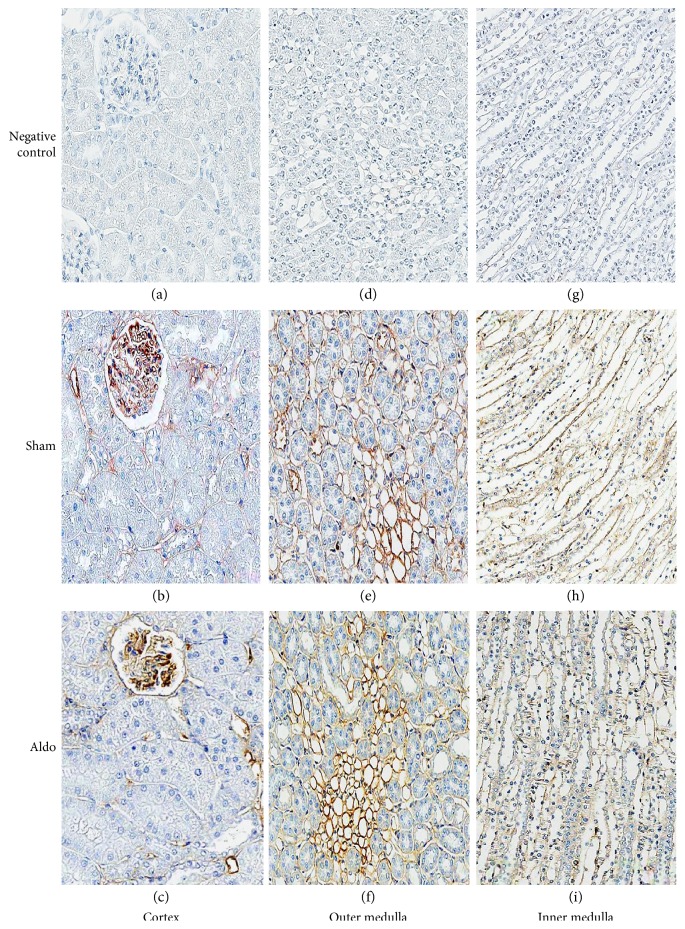
Representative immunohistochemical staining micrographs of renal PKC*β*II protein localization in the cortex (a–c), the outer medulla (d–f), and the inner medulla (g–i) from sham (b, e, and h) and Aldo (c, f, and i) (*n* = 5/group). Negative controls (a, d, and g). Original magnification, ×400 (a–c) and ×200 (d–i).

The protein localization of PKC*β*II in the cortex in the sham group is shown in Figure 4, b and Table 2. The immunoreactivity was moderate in the glomerulus and the Pcap, but no staining was noted in the PCT, the DCT, or the CCD. The immunoreactivity did not change by aldosterone in any studied area (Figure 4, c). In the OM, aldosterone elevated the intensity score from 2 to 3 in the tLH and staining in the TALH and the MCD diminished to trace levels. The intensity score in the VR remained moderate (Figure 4, f and Table 2). In the IM, immunoreactivity was lowered to trace levels in the MCD and the tLH by aldosterone (Figure 4, i).

### 3.6. Effect of Aldosterone on Renal NHE1 Protein Localization

**Figure 5:**
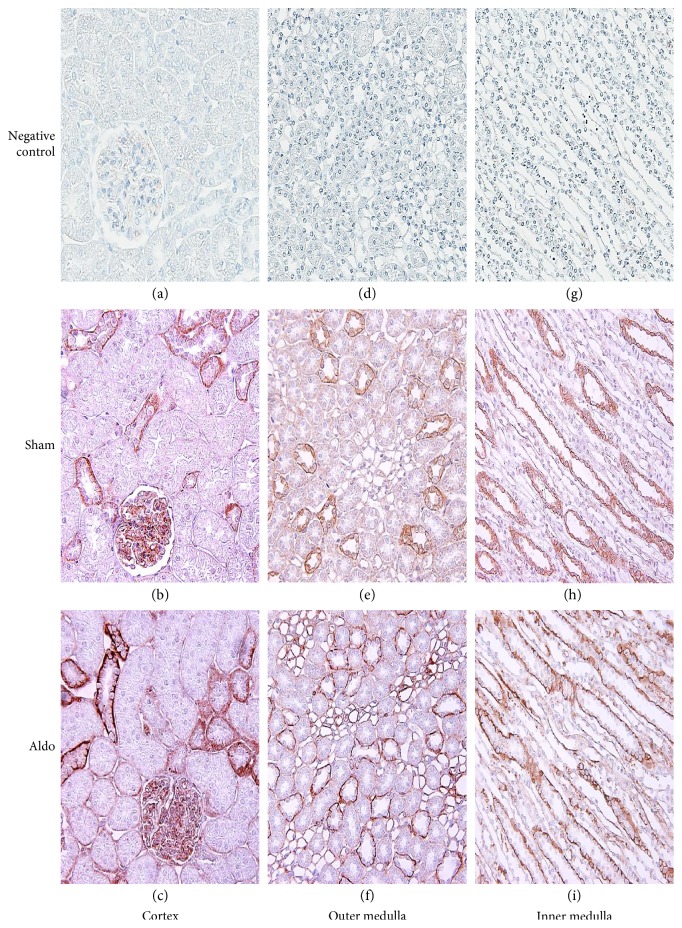
Representative immunohistochemical staining micrographs of renal NHE1 protein localization in the cortex (a–c), the outer medulla (d–f), and the inner medulla (g–i) from sham (b, e, and h) and Aldo (c, f, and i) (*n* = 5/group). Negative controls (a, d, and g). Original magnification, ×400 (a–c) and ×200 (d–i).

As shown in Figure 5, b and Table 2, in the cortex in the sham group, the immunoreactivity of renal NHE1 protein showed moderate staining in the glomerulus, trace diffused staining in the PCT, and prominent staining at the basolateral membrane of the DCTs and CCDs (Figure 5, b). Aldosterone increased the intensity score at the basolateral side of the PCTs and DCTs from 1 to 2 and in the CCD from 3 to 4 (Figure 5, c). The immunoreactivity in the glomeruli and the Pcap was unchanged by aldosterone. In the OM, aldosterone increased the staining to strong levels (score = 4) in the MCD, the VR, and the tLH, but the staining was unchanged in the TALH (Figure 5, f). In the IM, immunoreactivity was elevated in all studied areas by aldosterone (Figure 5, i and Table 2). Notably, in most of the studied renal tubular regions, aldosterone profoundly redistributed NHE1 protein from the cytosolic compartment to the basolateral membranes.

### 3.7. Effect of Aldosterone on Renal NHE3 Protein Localization

**Figure 6:**
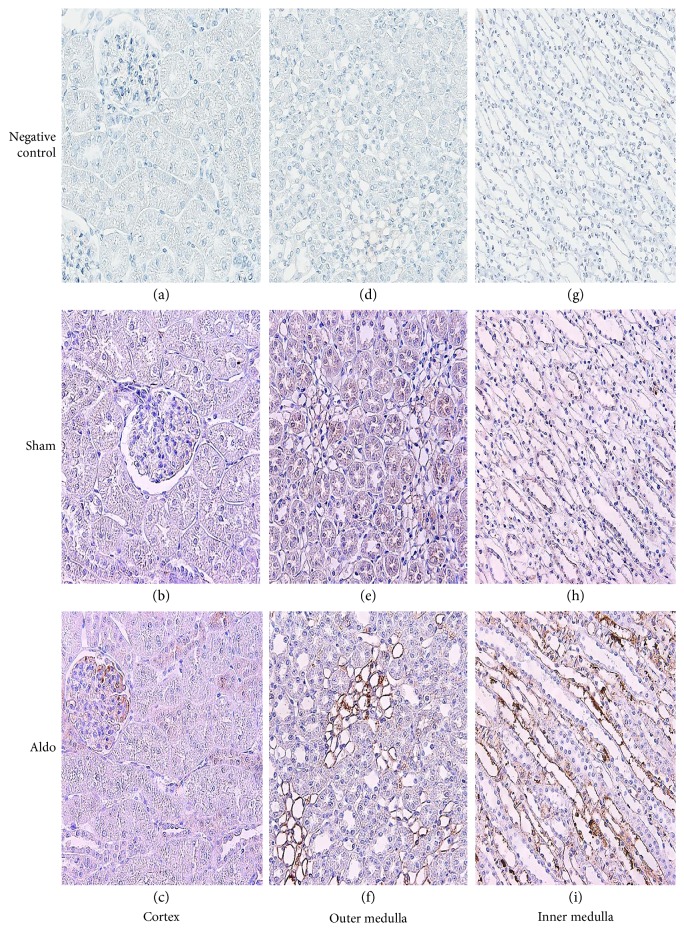
Representative immunohistochemical staining micrographs of renal NHE3 protein localization in the cortex (a–c), the outer medulla (d–f), and the inner medulla (g–i) from sham (b, e, and h) and Aldo (c, f, and i) (*n* = 5/group). Negative controls (a, d, and g). Original magnification, ×400 (a–c) and ×200 (d–i).

The protein localization of NHE3 in the cortex in the sham group is illustrated in Figure 6, b and Table 2. Immunoreactivity showed trace levels in all studied areas. Aldosterone slightly increased the intensity score to 2 only in the glomerulus and the CCD (Figure 6, c). In the TALH and the MCD of the OM, aldosterone diminished the intensity score from 2 to 1, but staining in the VR and the tLH was markedly elevated (Figure 6, f and Table 2). In the IM, aldosterone enhanced the immunoreactivity to a strong level in the VR and to a weak level in the tLH, while the intensity score was reduced in the MCD (Figure 6, i).

## 4. Discussion

The results in the present study provide the first *in vivo* data simultaneously showing renal PKC*β* (I and II) and NHE (1 and 3) protein abundance and immunoreactivity 30 minutes following aldosterone administration. According to Western blot analysis, aldosterone significantly enhanced the renal protein abundance of PKC*β*I by 30%, whereas PKC*β*II protein tended to decline (Figure 1). The rapid activation of the PKC family is a remarkable aspect of the rapid effects induced by aldosterone [20]. This may be due to aldosterone-induced transient increases in intracellular Ca^2+^ levels, which consequently activate Ca^2+^-dependent PKCs [10, 21]. A previous *in vitro* study in rat CCD cells showed that aldosterone administration for 15 minutes enhances the protein abundance of PKC*α* [10]. Moreover, we previously reported in the rat kidney that aldosterone injection for 30 minutes increased the protein abundance of PKC*α* and enhanced the immunoreactivity staining of PKC*α* in the PCT with the translocation from the basolateral to luminal membranes [11].

PKC*β* is a unique PKC isoform that encodes two proteins, PKC*β*I and PKC*β*II, generated by the alternative splicing of C-terminal exons [22]. There are no available data showing the rapid effect of aldosterone on this protein abundance. Our present data provide the first evidence that aldosterone rapidly activates PKC*β*I protein abundance in the rat kidney and that the protein level of PKC*β*II was maintained. This result implies that, by contributing its rapid effect, aldosterone shows a specific activation on PKC*β*I. The precise mechanism by which aldosterone enhances PKC*β*I protein abundance has not been established. PKC*β*I protein levels have been demonstrated to be enhanced within 5 minutes after incubation with reactive oxygen species [23]. Interestingly, aldosterone rapidly induced oxidative stress production [24, 25]. Therefore, we propose that the increased PKC*β*I protein abundance in the present study is mediated via oxidative stress induced by aldosterone.

For the immunolocalization of PKC*β*I and PKC*β*II, the baseline distribution of both isoforms is consistent with previous studies [26, 27]. The present data show that aldosterone increases the immunoreactivity of PKC*β*I protein in the glomerulus, the tLH, and the VR, but staining decreased in the PCT. For PKC*β*II, the immunoreactivity was maintained in the cortex area. However, the staining in the medullary region was altered differently. Aldosterone enhanced the PKC*β*II immunoreactivity in the tLH of the OM, but decreased the immunostaining in the IM and staining in the TALH and the MCD declined. The present results indicate that, in a rapid pathway, aldosterone diversely regulates the protein distribution of PKC*β*I and PKC*β*II along the nephron. These proteins can consequently modulate myriad renal tubular ion transport [28, 29]. Previous studies demonstrated that PKC plays important roles in the maintenance of normal cellular functions, mediates the repair of mitochondrial and transport functions after toxicant-induced injury, and regulates cell survival in renal proximal tubular cells [30, 31]. This suggests a significant action of aldosterone on tubular functions via PKC.

Notably, the activation of PKC*β* seems to contribute to the pathophysiological deflection of renal tissues [32, 33]. A previous *in vitro* study demonstrated that the overexpression of PKC*β*I and/or PKC*β*II induced fibronectin production, increased transforming growth factor beta 1 release, and caused morphological changes in human proximal tubular epithelial cells [34]. These alterations were completely blocked by a PKC*β* inhibitor [34]. Moreover, treatment with a PKC*β* inhibitor in diabetic db/db mice diminished the accelerated diabetic mesangial expansion [35]. Furthermore, the deletion of PKC*β* (PKC*β*^−/−^) decreased oxidative stress, cytokine expression, renal dysfunction, and hypertrophy in a streptozotocin-induced diabetic mouse model [36–38]. Collectively, PKC isozymes contribute to diverse cellular responses [39].

For NHE1, in the present study (Figure 2()), the administration of aldosterone for 30 minutes increased protein abundance by 52% (*P* < 0.05). NHE1, located at the basolateral membrane of most nephron segments, extrudes one proton in exchange for one sodium ion entering into the cells to regulate cellular pH and volume homeostasis [40–43]. No data are available regarding the rapid effect of aldosterone on NHE1 protein abundance. Most previous investigations have documented the rapid action of aldosterone on NHE1 activity [7, 9, 44–46]. For example, exposure to aldosterone (2–20 minutes) stimulated NHE1 activity and increased intracellular pH [pH]_i_ in isolated proximal S3 segments, giant cells fused from individual target cells of the distal nephron of the frog kidney, and MDCK cells [9, 44–46]. These alterations induced by aldosterone were inhibited by an NHE blocker [44]. Consistent with hydrogen efflux activated by NHE, aldosterone rapidly induced sodium influx by more than 10-fold and increased MDCK cell volume [47]. For NHE1 localization, the immunostaining of NHE1 protein in the sham group revealed a similar baseline regional distribution as previously described [40, 41]. The present data show that aldosterone rapidly induces a greater amount of NHE1 protein immunoreactivity in the medulla region than in the cortex, with profound redistribution of NHE1 protein from the cytosolic compartment to the basolateral membrane (Figure 5()). This result implies that rapid effects of aldosterone have important roles in the regulation of renal tubular function.

NHE3, localized at the apical membrane mostly in the PT and the TALH, expels one hydrogen ion in exchange for one sodium ion entering into the cells [48–50]. NHE3 has an influential role in bicarbonate reabsorption, sodium and volume homeostasis, and in the reabsorption of other substances by functionally coupling to a variety of other transporters [50]. The present study shows that aldosterone rapidly enhances NHE3 protein abundance by 34% (*P* < 0.05) (Figure 2). A previous study demonstrated that aldosterone stimulates NHE3 trafficking and increases NHE3 surface expression [7]. In contrast, in the renal medullary thick ascending limb (mTAL), aldosterone rapidly decreased bicarbonate reabsorption in an MR-independent manner [51]. This effect was not inhibited by pretreatment with transcription or translation blockers [51]. Moreover, in the mTAL, aldosterone reduced bicarbonate reabsorption within 15 minutes by inhibiting apical NHE3 activity [52]. The inhibition remained, despite blocking NHE1 function, and was preserved in the mTAL in NHE1 knockout mice [52]. For NHE3 localization, the baseline distribution of NHE3 protein is consistent with previous studies [48–50]. Our present results show that aldosterone increases more NHE3 protein immunostaining in the medulla area than in the cortex. Interestingly, aldosterone reduced NHE3 immunoreactivity in the mTALH (Figure 6, Table 2). This result indicates that aldosterone diminishes bicarbonate reabsorption in the mTALH not only by suppressing apical NHE3 activity but also by reducing NHE3 protein immunoreactivity.

It has been shown that stimulated PKC can influence NHE activity. A previous study in a luminal membrane vesicle isolated from rat kidney cortical tubules showed that PKC activation by phorbol myristate acetate for 4 minutes increases NHE3 activity, with no change in protein abundance [53]. The explanation for these PKC-induced alterations may result from the activation of a regulator component of NHE3 [53]. In M-1 CCD cells, aldosterone caused a rapid increase in [pH]_i_ recovery through the activation of NHE1, which was attenuated by a PKC inhibitor but not by an MR antagonist [6].

Another attractive mechanism by which aldosterone enhances NHE properties is aldosterone-transactivated epidermal growth factor receptor (EGFR) and extracellular signal-regulated kinase 1 and 2 (ERK1/2). It has been shown that NHE activity can be controlled by ERK1/2 [54]. ERK1/2 induced by aldosterone can dependently phosphorylate and then activate NHE1 in CCD cells [6]. In MDCK cells, aldosterone rapidly induced NHE activity, cellular alkalization, and ERK1/2 phosphorylation [55]. These were prevented by the administration of an ERK1/2 inhibitor [55]. We previously demonstrated that aldosterone rapidly elevates EGFR phosphorylation and activates ERK1/2 in the rat kidney [13]. Therefore, in the rat kidney, the rapid effect of aldosterone on NHE activation might be mediated through PKC and/or EGFR-ERK1/2 cascade stimulation.

## 5. Conclusion

This is the first *in vivo* study to simultaneously demonstrate that aldosterone rapidly enhances PKC*β*I and NHE (1 and 3) protein levels in the rat kidney. Aldosterone-induced NHEs protein abundance and immunoreactivity may be mediated through PKC*β*I activation.

## Figures and Tables

**Table 1:** Blood and urine chemistry in experimental groups.

	Sham	Aldo
Plasma aldosterone (pmol/L)	1315.11 ± 10.22	6234.33 ± 92.88^∗^
Plasma sodium (mmol/L)	141.32 ± 1.48	142.17 ± 6.32
Plasma potassium (mmol/L)	3.53 ± 0.22	3.58 ± 0.26
Plasma chloride (mmol/L)	102.64 ± 1.34	102.31 ± 2.91
Plasma bicarbonate (mmol/L)	24.71 ± 1.14	24.29 ± 1.58
Plasma creatinine (mg/dL)	0.24 ± 0.02	0.24 ± 0.02
Blood urea nitrogen (mg/dL)	19.22 ± 1.64	20.48 ± 3.28
Ratio of plasma sodium to potassium	41.27 ± 3.64	39.87 ± 2.94
Ratio of urinary sodium to potassium	0.32 ± 0.02	0.32 ± 0.02

Data are expressed as means ± SD, *n* = 8/group; ^∗^*P* < 0.001 compared with the sham group.

**Table 2:** Median staining intensity (score) of renal PKC*β*I, PKC*β*II, NHE1, and NHE3 protein localization.

	Median staining intensity (score)							
	PKC*β*I		PKC*β*II		NHE1		NHE3	
	Sham	Aldo	Sham	Aldo	Sham	Aldo	Sham	Aldo
Cortex								
Glomerulus	2	3	3	3	3	3	1	2
PCT	0	0	1	1	1	2	1	1
DCT	0	0	0	0	1	2	1	1
CCD	0	0	0	0	3	4	1	2
Pcap	2	3	3	3	2	2	1	1

Outer medulla								
TALH	1	1	2	1	1	1	2	1
MCD	1	1	3	1	3	4	2	1
VR	3	4	3	3	1	4	2	4
tLH	3	4	2	3	1	4	2	4

Inner medulla								
MCD	1	1	3	1	3	4	1	0
VR	1	2	1	1	1	2	1	4
tLH	1	2	2	1	1	2	1	2

Staining intensity: 0 = negative, no reactivity; 1 = trace, faint or pale brown staining with less membrane reactivity; 2 = weak, light brown staining with incomplete membrane reactivity; 3 = moderate, shaded of brown staining of intermediate darkness with usually almost complete membrane reactivity; 4 = strong, dark brown to black staining with usually complete membrane pattern, producing a thick outline of the cell [11–13]. PCT: proximal convoluted tubule; DCT: distal convoluted tubule; CCD: cortical collecting duct; Pcap: peritubular capillary; TALH: thick ascending limb of the loop of Henle; MCD: medullary collecting duct; VR: vasa recta; tLH: thin limb of the loop of Henle (*n* = 5/group).
